# Development of a cell-free protein synthesis system for practical use

**DOI:** 10.2183/pjab.97.015

**Published:** 2021-05-11

**Authors:** Yaeta ENDO

**Affiliations:** *1 Ehime Prefectural University of Health Sciences, Tobe-cho, Iyo-gun, Ehime, Japan.

**Keywords:** ribosome, ribotoxin, wheat embryos, cell-free protein synthesis

## Abstract

Conventional cell-free protein synthesis systems had been the major platform to study the mechanism behind translating genetic information into proteins, as proven in the central dogma of molecular biology. Albeit being powerful research tools, most of the *in vitro* methods at the time failed to produce enough protein for practical use. Tremendous efforts were being made to overcome the limitations of *in vitro* translation systems, though mostly with limited success. While great knowledge was accumulated on the translation mechanism and ribosome structure, researchers rationalized that it may be impossible to fully reconstitute such a complex molecular process in a test tube. This review will examine how we have solved the difficulties holding back progress. Our newly developed cell-free protein synthesis system is based on wheat embryos and has many excellent characteristics, in addition to its high translation activity and robustness. Combined with other novel elementary technologies, we have established cell-free protein synthesis systems for practical use in research and applied sciences.

## Introduction

The concept of cell-free protein synthesis is based on the idea of carrying out translation reactions in a test tube using the translation machinery extracted from living cells. In 1961, Nirenberg and colleagues reported mRNA-dependent polypeptide synthesis using an extract from *Escherichia coli*.^1)^ This cell-free system enabled deciphering of the genetic code. Furthermore, the method revealed a full picture of the translation mechanism and opened the door to molecular biology as we know it today. Because the translation apparatus in a cell-free system retains the activity, speed, and accuracy of polypeptide synthesis present in living cells, such a system can be used as an excellent and essential analytical tool. However, the productivity of these conventional systems was too low, and protein products were only detected by incorporation of radio-labeled amino acids in tracer experiments. Higher protein yields were thought to be unachievable because; 1) low productivity had been generally observed in all reported systems; and 2) it was impossible to work on a high throughput for analyzing hundreds of transcribed elements in a test tube. Therefore, the idea of robust protein synthesis in a cell-free system was essentially dismissed. As a graduate student in the 1970s, while seeking to understand the structure and function of the ribosome, I had often thought about potential ways to overcome the above-mentioned limitations of classical cell-free systems.

**Figure 1.**
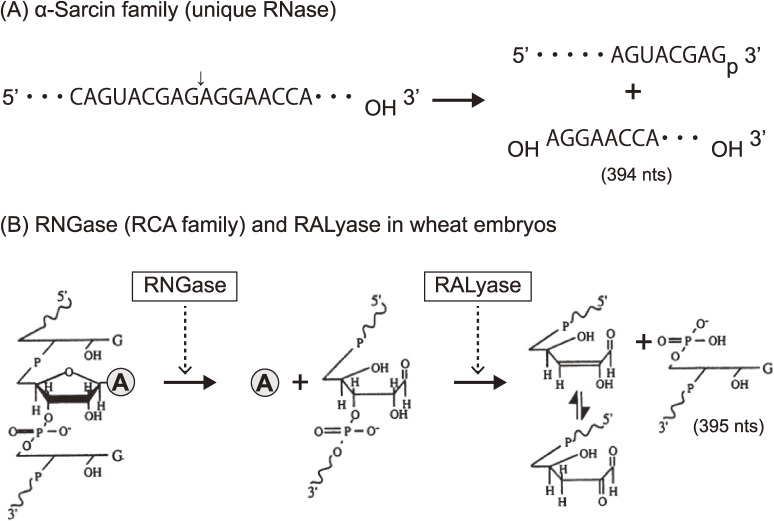
Modes of action of two families of RIPs. (A) α-Sarcin catalyzes the hydrolysis of one phosphodiester bond (indicated with an arrow) between G_4325_ and A_4326_ in rat 28S rRNA and produces an α-fragment (3′ side of 28S, 394 nucleotides). (B) Chemistry of RNGase- and RALyase-catalyzed reactions. RNGase hydrolyzes an adjacent *N*-glycosidic bond at A_4324_ in the same loop as shown in (A), releasing adenine from ribosomes. RALyase in wheat embryos cleaves the phosphate backbone at the apurinic site through its lyase activity, yielding a fragment of 395 nucleotides containing the 3′-end with a 5′-terminal guanosine-5′-phosphate residue. A simple depurination assay (RNGase activity) can be done by means of gel-electrophoresis to see the formation of the fragment (395 nucleotides) after the treatment of extracted RNA with acid-aniline.^7,8)^

**Figure 2.**
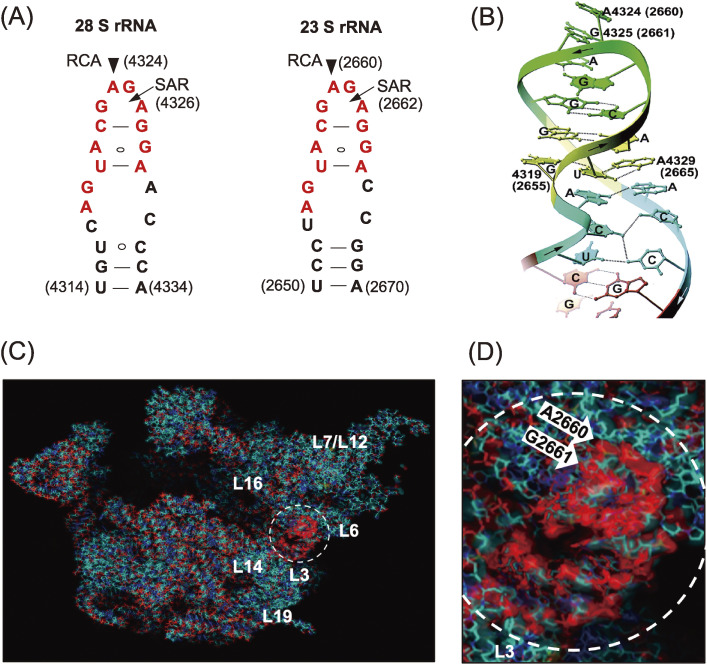
Sites of action of SAR and RCA in a conserved nucleotide sequence (SRL) in rRNAs and their structures. (A) The sites on 2D Watson–Crick base-pair models. Arrowheads and arrows mark RCA and SAR sites, respectively, in the universally conserved nucleotide sequence (highlighted in red color). (B) Schematic representation of the X-ray crystal structure of a synthetic 29-mer of rat SRL.^17)^ (C) The crystal structure of *E. coli* 50S subunits and the SRL (dotted circle) is shown with a magnified view (D). Ribosomal proteins and RNA-chain, including SRL, are marked in blue and red, respectively. Copyright (1998) National Academy of Sciences, U.S.A. (for Fig. 2A and B). Figure 2C and D are a gift from H. F. Noller, who kindly created the picture based on the published databank.^20)^

Ribosomes are known as large molecular machines (a complex of ∼80 proteins (r-proteins) and 4 RNAs (rRNA) in eukaryotes, and ∼55 r-proteins and 3 rRNAs in prokaryotes) which catalyze polypeptide synthesis with accuracy and high speed. However, the structure and function of ribosomes were largely elusive in those days. Nomura and colleagues made great strides in ribosome research using their reconstitution technique to determine which moiety, the proteins or the RNA, might contribute the major function in protein synthesis. They successfully reconstituted fully active hybrid 30S subunits by assembling *E. coli* 30S r-proteins and 16S rRNA from phylogenetically distant *Bacillus subtilis*. Moreover, they found that introduction of multiple nicks into the rRNA in *E. coli* ribosomes did not affect any activity. Based on their findings, it was concluded that the key players in the ribosomes were the r-proteins rather than rRNA.^2)^ Although lines of genetic and biochemical evidence for mechanisms of action for some antibiotics had suggested that their targets were on rRNA, these antibiotics were thought to inhibit ribosomal function by disturbing the scaffold formed by the rRNA chain leading to improper arrangement of the functional r-proteins. With this idea, Wittmann and colleagues made considerable efforts to find r-proteins that carry out any elementary function of ribosomes.^3)^ Around that time, we had started investigating the mechanisms of action of ribotoxins, known as ribosomal inactivating proteins (RIPs), in order to understand the structure and function of ribosomes. One of such RIP, α-Sarcin (SAR), is a cytotoxic protein originally found as an anticancer protein isolated from cultured *Aspergillus giganteous*. SAR was known to inhibit protein synthesis enzymatically by inactivating ribosome functions during peptide formation, and we elucidated the mode of action of this RIP. We found that SAR is a ribonuclease (RNase) that specifically hydrolyzes a single phosphodiester bond between G_4325_ and A_4326_ in an evolutionally conserved sequence at the 3′-end of eukaryotic 28S rRNA (or in 23S rRNA in bacteria). This produced a 394-nucleotide fragment (α-fragment) with a hydroxyl group at the 5′-end of the downstream rRNA (Fig. 1A).^4–6)^ We also described another type of ribotoxin, ricin A-chain, found in the seeds of castor beans, *Ricinus communis*. Despite the beautiful flower it comes from, the protein is known as a deadly toxin. The A-chain of ricin (RCA) enzymatically inactivates the elongation-factor (EF-1 and EF-2)-dependent transpeptidation reaction of ribosomes, whereas the B-chain is a lectin that delivers RCA into animal cells where the inactivation of ribosomes takes place. Although the molecular mechanism of action of RCA had been unknown for a long time, ricin had been used for assassinations because of its deadly toxic action, where tens of micrograms were enough to kill a human. We investigated the mode of action of RCA and successfully solved the mystery of the mechanism.^7–11)^ The target site of RCA was determined to be A_4324_ in the rRNA, adjacent to the specific cleavage site by SAR, and it was shown that the protein catalyzes the hydrolysis of the N-glycosidic bond between adenine and ribose. The catalytic mechanism was unique, and we named this enzyme an “RNA N-glycosidase” or RNGase for short (Fig. 1B). Meanwhile, we also found that the A-chains of both Shiga toxin and Vero toxin, had an identical mode of action and functioned in the same way as RCA.^12,13)^ Thus far, two distinct families of ribotoxins irreversibly inactivate the peptide elongation activity of ribosomes solely by hydrolyzing a single phosphodiester bond (SAR) or performing a single deadenylation (RCA) at one contiguous site among ∼7,000 nucleotides in eukaryotic ribosomes (or ∼5,000 nucleotides of in *E. coli* ribosomes). Kinetic parameters of RCA activity on ribosomes were analyzed, with apparent *K*m and *K*cat determined to be ∼2 µM and ∼2,000 s^−1^, respectively.^9)^ It has been shown that about 15 ribosomes on average are functioning on a single mRNA forming polysomes in translationally active cells. Upon inactivation of peptide elongation by a RCA, freezing of ribosomes takes place on polysomes, which physically blocks the movement of all following intact ribosomes. Thus, in theory, the depurination (inactivation) of about 7% of the entire ribosome population in a cell is enough to completely terminate protein synthesis, thus leading to cell-death, which turned out to be the reason for the extreme toxicity of ricin to humans. As shown in Fig. 2A, both SAR and RCA recognition-sites are in the same small stem-loop on the specific 2-dimensional (2D) Watson–Crick structure model consisting of 12 nucleotides (indicated in red). This sequence is universally conserved among organisms. We named this small, but known to be the longest conserved nucleotide sequence in rRNA, the “Sarcin-Ricin Loop” (SRL). Our findings suggested that the SRL has a structurally and functionally important role in ribosomes.^14)^ The 3D structure of SRL in the naked form and in ribosome particles was determined using NMR^15,16)^ and X-ray crystallography.^17)^ Figure 2B shows the structure of a synthetic 29-mer representing the eukaryotic SRL, as determined using X-ray crystallography. The adenosine residue, the target nucleotide of the RNGase, sticks out from the helix, making it more accessible. A matching GAGA, tetra-nucleotide loop, and five base pairs formed practically the same 3′ structure in the structure of 50S ribosomes, as solved by X-ray crystallography (Fig. 2B and D), thus confirming our results obtained with a synthetic oligonucleotide. Noller and others pointed out that the SRL plays a central role for two elongation factors, thereby triggering the dynamic structure of the 50S unit in functioning 70S ribosomes.^17,18)^ Together with these findings and others, it is now widely accepted that “The ribosome is a ribozyme”.^19–21)^ However, in those days, we were asking, “What is the biological significance of RNGase in nature?” To obtain clues to answer this question, we first surveyed the distribution of RNGase among plants^22–25)^ using a simple acid-aniline assay, which we had developed. From the initial screening, we demonstrated that the RIPs and some of the so-called antiviral proteins were almost ubiquitously distributed within the plant kingdom, including grains. However, it should be noted, most of these single-chain ribotoxins lacked the B chain, so they are not toxic to animals. Using immunoelectron microscopy, RNGases, including the pokeweed antiviral protein, were found to be localized in the extracellular space of the cotyledon.^26–28)^ Moreover, we found another novel enzyme, a ribosomal RNA apurinic site-specific lyase (RALyase), in wheat embryos, which cleaved a phosphodiester bond at the apurinic site within 28S rRNA in ribosomes, producing a 395 nucleotide fragment leaving a 5′ phosphate in the rRNA (Fig. 1B).^29)^ Deadenylated ribosomes have little activity in the two elongation factor-dependent translation reaction, as mentioned above. However, they showed full peptide synthesis activity with ribosomes fully possessing poly-phenylalanine synthesis activity when measured in artificial conditions using high magnesium ion concentrations in the absence of the above-mentioned elongation factors. Those ribosomes, however, were totally inactivated once the chain scission was introduced by RALyase,^13,29)^ just as SAR-treated ribosomes were. These finding suggested that there might be a cooperative role between tritin (wheat RNGase later), localized in the outer space of cells, and RALyase inside the embryos to achieve the termination of ribosome function. Considering the functions of RNGase and RALyase, their cellular localization, and the antiviral action altogether, we speculated that the system might be functioning as a plant self-defense mechanism through killing their own ribosomes to stop viruses. In this scenario, once the cuticle of the plant cell wall is damaged by insect bites, RNGase flows into the cytoplasm of embryonic cells and cleaves the N-glycosidic bond of ribosomes. The depurinated ribosomes are then completely terminated by RALyase in embryonic cells, thus preventing the proliferation of viruses. Hence, we speculated that the programmed suicidal system might have evolved together with the translation system not only in the plant kingdom but may also be used as a general defense system. Such antiviral self-defensive mechanisms are well known in animals based on research on interferon.^30)^

Having this defense mechanism in mind, we reconsidered the reasons for the common observation of “the low productivity of classical cell-free systems”. We hypothesized that the low productivity of reported systems may, in part, be caused by an artificial event during the homogenization of the raw materials, which would induce a malfunction of the self-defensive system, resulting in killing of the translation machinery. The most well-studied plant-based cell-free protein synthesis system was the wheat germ cell-free system originally reported-by Roberts and colleagues. This wheat germ cell-free system had the highest translation activity with a prolonged reaction time of an hour, whereas the *E. coli* system worked only for 10–15 min.^31)^ We expected that the wheat system might be further improved by eliminating the self-defensive system and other endosperm-originating contaminants. Dormant embryos are known to store a large amount of the translation machinery for upcoming germination, although tritin, a single-chain RIP,^32)^ was also reported in wheat seeds and leaves. Initially, it was reported in a functional analysis that wheat embryonic ribosomes are resistant to tritin.^32–34)^ However, we directly demonstrated that tritin indeed possesses a depurination activity at A_4324_ of the 28S rRNA of embryonic ribosomes. Furthermore, we were able to solve this problem by simply washing crude embryos before the homogenization step during wheat extract preparation. The effect was far more successful than we expected, and this became the first essential point toward a practical cell-free expression system described in the later section.

The second crucial point we addressed was the robustness of the reaction, a mode of the translation reaction set up to meet the needs of a practical use. Spirin and colleagues had been working on improving the productivity of cell-free systems mainly by focusing on the reaction mode. They believed that the short reaction time, which was generally observed for *in vitro* translation reactions, must be caused by the consumption of substrates (amino acids, ATP, GTP) during the reaction. Therefore, they introduced a continuous-exchange cell-free (CECF) reaction method to replace the commonly used regular batch-wise reaction, which significantly improved the capability of their new system.^35)^ However, their original CECF method, using an ultra-filtration membrane, was not very reproducible mainly due to clogging of the membrane, and thus their method could not be fully utilized. However, it was fortunate that we were able to reproduce a part of their protocol.^36)^ Later, we applied the two different reaction procedures, using the dialysis reaction mode for large-scale production and a bilayer reaction mode for the parallel production of small protein quantities.

Inevitably, the quality of synthesized proteins is critical, and the ability to produce a properly folded active protein has been the third essential need for a practical system. The folding mechanisms for newly synthesized proteins are entirely different in prokaryotes and eukaryotes, which seemed to be coupled with the speed of peptide bond formation, where the rate in *E. coli* is about 10 times faster than that in eukaryotic cells. It was assumed that protein folding in bacterial cells mainly takes place post-translationally with the help of chaperones, whereas in eukaryotes co-translational folding plays a key role in protein folding with the help of partner molecules such as prosthetic groups.^37,38)^ As a result, the *E. coli*-based expression methods, whether *in vivo* or *in vitro*, undoubtedly have limitations in synthesizing eukaryotic proteins in their active forms, especially for larger polypeptides commonly found in eukaryotes. In fact, it is quite often observed that the products are segregated as an insoluble inclusion body, when eukaryotic proteins are expressed in *E. coli* cells. In this respect, a wheat-based expression system can better address co-translational folding because of its lower translation speed. In addition, our wheat cell-free technology may solve other problems, such as biohazardous, ethical issues, and cost-effectiveness. It was fortunate for us that we had chosen the wheat platform for improving *in vitro* translation systems.

Hereafter, this review will introduce the elementary technologies that we have developed; 1) preparation of a highly efficient and robust extract from wheat embryos, 2) an optimized mRNA design, 3) customized DNA template design for *in vitro* transcription, 4) a platform for a simple CECF mode translation reaction, and 5) the world’s first fully automated protein synthesizers were invented combining all these technologies into a comprehensive system.

## 1 Preparation of an extract with a high translation activity from purified wheat embryos.

### (1) Preparation of a high-quality extract.

**Figure 3.**
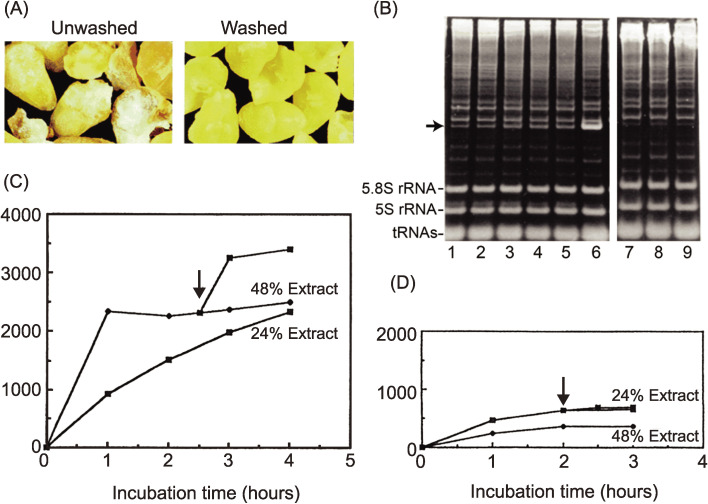
Robust protein synthesis using wheat extract prepared from washed wheat embryos. (A) Extracts (S30, 240 A_260_ OD units/ml) were prepared from unwashed and washed embryos, respectively. (B) The depurination assay used to monitor tritin activity in unwashed or washed embryos. Translation mixtures were constructed with extracts from unwashed or washed embryos and were incubated in a batch-wise reaction for the indicated times, and phenol-extracted RNA samples from each time point were treated with acid-aniline to cleave the phosphate backbone at the depurinated site. Lanes 1–5 contain samples after incubation of the mixture with unwashed embryos for 0, 1, 2, 3, and 4 h, and lanes 7–9 contain samples after incubation of the mixture with washed embryos for 0, 2, and 4 h, respectively. Lane 6 shows the reaction marker, in which a fragment was produced by incubation in the presence of gypsophilin, a highly active RNA N-glycosidase from *Gypsophila elegance*.^28)^ The arrow indicates the aniline-induced fragment of the 3′-side of wheat 28S rRNA. (C) Protein synthesis capacities of an extract from washed embryos using the DHFR mRNA with a 5′-Cap structure and 100-nucleotide poly(A) tails, transcribed from pSP65 plasmid. Translation reactions were carried out in test tubes for the indicated time course. Protein synthesis was measured using hot-trichloroacetic acid-insoluble radioactivity of incorporated ^14^C-labeled leucine. (D) The same experiment was carried out as in (C) using an extract from unwashed embryos. Arrows indicate the time points of supplementation of the substrate solution.^49)^ Copyright (2002) National Academy of Sciences, U.S.A.

As shown in Fig. 3A, whitish powder (wheat flour) could be seen on the surface of crude embryo preparations, which indicated that isolated embryos from the seeds are contaminated with the remaining components of the endosperm. The extract was directly prepared, and cell-free translation reactions were performed in batch-wise reactions following the protocol reported by Robert and others.^31)^ We began by examining the occurrence of any depurinated ribosomes caused by contaminating tritin using the acid-aniline assay and found that the number of depurinated ribosomes increased overtime, as shown by the formation of a 385-nucleotide fragment on the gel (arrow in Fig. 3B, lanes 1–5). Furthermore, even before the incubation, about 7% of the ribosome population had already been depurinated. When RNA was directly extracted from embryos using the guanidine isothiocyanate-phenol method, a harsh condition to denature tritin and other proteins, little formation of the aniline-induced fragment was observed, indicating that the depurination reaction had already taken place during the extraction process. Because we knew that even a small portion of depurinated ribosomes led to severe inhibition of protein synthesis, we decided to remove tritin from the extract. Attempts were made to neutralize the enzyme with chemical components, including synthetic RNA aptamers that bound tightly to the RIP,^39)^ but these approaches did not work. Instead, careful selection and subsequent extensive washing of isolated embryos resulted in an excellent result. The depurination assay showed an undetectable level of fragment formation in the acid-aniline assay (Fig. 3B, lanes 7–9), indicating that these washed embryos contained little tritin, and other contaminants had probably also been removed. As shown in Fig. 3C and D, the cell-free system prepared from washed embryos had much higher translation activity than the conventional system.^40)^ When programmed with an mRNA encoding dihydrofolate reductase (DHFR) as a model, the reaction continued for at least 4 hours in a system containing 24% extract (Fig. 3C), as opposed to the original system (unwashed), which ceased activity after 1.5 hours (Fig. 3D). In the reaction containing 48% of the new extract, the initial reaction rate was increased two-fold compared with that using 24% of the extract; however, the translation reaction stopped after 1 hour. Halting of the reaction (arrows) was overcome with kinetics similar to the initial rate after supplementing fresh substrate, including amino acids, ATP, and GTP. In contrast, 48% of the extract from unwashed embryos showed even lower activity than 24% of the extract, and the addition of fresh substrate after the reaction had stopped did not have any effect (Fig. 3D). These observations were consistent with the results of the above-mentioned depurination assays, indicating that there was irreversible damage done to the ribosomes by tritin and probably other contaminants such as RNase(s) from the endosperm. A high translation activity of the system with washed embryos was also demonstrated using a sucrose density gradient experiment, in which polysome formation can be monitored. Significant numbers of newly formed polysomes were observed after incubation for 1 hour, and a shift to heavier polysomes with a concomitant decrease in 80S monosomes was found after incubation for 2 hours.^40)^ Moreover, electron microscopic examination revealed, for the first time, *in vitro* formation of circular-shaped polyribosomes, which are well known from living cells with high protein synthesis activity.^41)^ There is an additional explanation for the dramatic improvement in protein synthesis after washing of the embryos. Thionins are a group of small, basic, cysteine-rich proteins, originally purified as antifungal proteins from various plants, including wheat seeds.^42)^ Wheat γ-thionin is known to be in the endosperm of seeds,^43)^ and Brummer and colleagues showed that α- and β-thionins from barley endosperm are potent inhibitors of protein synthesis initiation in a wheat germ translation system.^44)^ In addition, several nucleases have been reported in the endosperm of seeds.^45)^ Thus, it was thought to be possible that washing of the embryos also resulted in the elimination of thionin and ribonucleases together with tritin.

### (2) Performance of the new extract.

After establishing a procedure for the preparation of highly active wheat embryo extract, we examined its possible application for protein production. For this purpose, we adapted a dialysis system as a simplified CECF mode translation reaction. With capped mRNA encoding DHFR and with a poly(A) tail, protein synthesis worked efficiently. The reaction proceeded for up to 60 hours, yielding 4 mg of active enzyme in a 1 ml reaction when supplemented with fresh mRNA every 24 hours.^40)^ This result directly indicated the robustness of the system and its high translational activity. In theory, the strategy we have introduced for making our system work is applicable to other cell-free systems; however, it may not be a trivial task. For example, an RNase1 deletion mutant has been used for constructing an *E. coli* cell-free system, though there is still a non-negligible amount of RNase 1 together with other cellular RNase activities in the extract. Rabbit reticulocyte lysate also contains RNase M attached to the plasma membrane, which cannot be eliminated by any available procedure. These RNases, even occurring at low concentrations in the translation mixture, bring the system a drawback to complete translation reactions for high molecular weight proteins because the mRNA template will always be extremely sensitive to RNases. Thus, it is hard to expect any formation of large, circular polysomes in these systems. In general, to optimize the reconstitution of any given biochemical reaction *in vitro*, limiting factors must be found and removed from the artificially created systems. Hence, we carefully optimized each of the ingredients and the translation conditions, including the incubation temperature for the new extract.

## 2 Development of other elementary technologies for improving the wheat germ cell-free protein synthesis system.

### (1) Designing an efficient mRNA and construction of a DNA template for *in vitro* transcription.

The 5′-Cap (7-mG-5′-ppp-5′G) plays a crucial role in eukaryotic translation initiation, but it was initially problematic in cell-free translation systems. When RNA templates are prepared *in vitro*, the cap structure can be introduced using an RNA polymerase that incorporates the modified free dinucleotide at the 5′-end of an RNA polynucleotide chain. However, the efficiency of this incorporation was generally low, and there was inevitably an excess of free modified nucleotide remaining in the reaction mixture. Moreover, the free modified nucleotide binds competitively to the cap-binding protein, eIF4E, thereby inhibiting translation. Complete removal of the free nucleotide is tedious and not easily accomplished in the context of a high-throughput system. Furthermore, there is a very narrow range for suitable concentrations of capped RNA that give efficient translation; this range is best determined empirically because the exact concentration of effectively capped RNA is difficult to determine.^40)^ This optimization is problematic when proteins are to be synthesized from many genes (RNAs) in parallel. The poly(A) tail at the 3′-end of mRNA is also a problem when preparing templates for *in vitro* translation reactions because plasmids with long poly dT/dA sequences are unstable during replication in *E. coli* cells. To solve these problems, we developed new 5′- and 3′-UTRs (untranslated regions) that enhance mRNA translation in the absence of a cap structure and a poly(A) tail.

**Figure 4.**
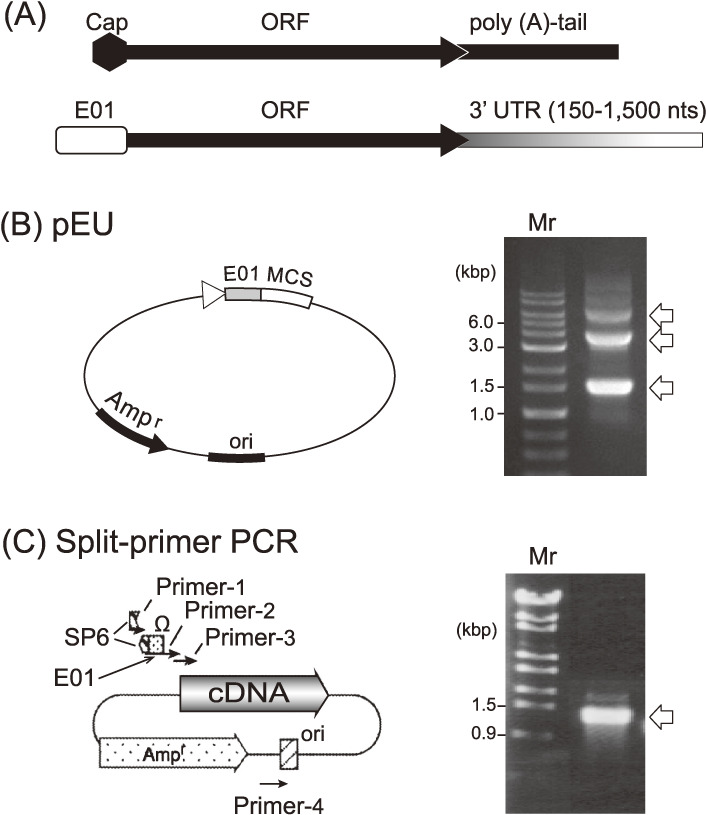
Two methods to construct DNA templates for *in vitro* transcription. (A) 5′- and 3′-end structures of a typical eukaryotic mRNA (*upper row*) and the newly designed one (*lower*) are shown. (B) The plasmid, originating from pSP65, possesses an SP6 polymerase promoter, a newly developed enhancer sequence (E01), multi-cloning sites, and a replication site (Ori) (*left*). Circular pEU carrying a luciferase gene with E01 gave 3 mRNA bands when transcribed (arrows on the gel) (*right*). (C) A split-primer PCR method was developed for the parallel transcription of cDNAs from a cDNA library, minimizing primer-dimer byproducts (*left*). A set of primers in which the complete promoter sequence was not contained in any one of the primers but was generated only when the sequences of two primers were joined correctly, leading to the production of only complete mRNA (arrow, *right*).^49)^ Copyright (2002) National Academy of Sciences, U.S.A.

**Figure 5.**
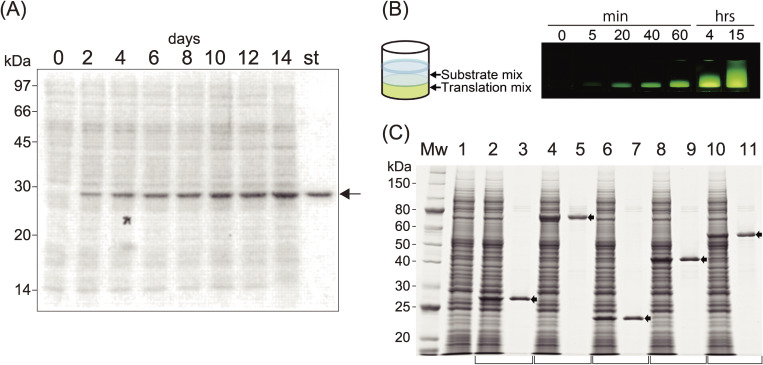
Performance of a cell-free system. (A) SDS-PAGE analysis of GFP produced during reactions lasting for 14 days. mRNA produced by transcription of circular pEU was used for the translation reaction in a dialysis membrane system and was added every 48 h. A 0.1 µl aliquot of the mixture was run on a gel, and protein bands were stained with CBB. The arrow shows GFP, which appears as a prominent band among the endogenous protein bands (lanes, 0–14 days). “st” designates a 0.5 µg equivalent of an authentic GFP band. (B) In small-scale protein synthesis, the translation solution was layered under the buffered substrate mixture. The two solutions (usually 25 µl on the bottom and 125 µl on the top in a 96 microtiter plate, the bilayer mode reaction) mix together gradually during incubation period, enabling continuous substrate supply and dilution of byproducts, which may otherwise suppress the translation reaction (*left*). The translation reaction continued for about 20 h at 15 ℃, and time-dependent synthesis of GFP and diffusion of two layers are shown (*right*). (C) Five mRNAs, transcribed from PCR-generated DNA templates with designed C-term His-tag sequences, were translated overnight. The crude reaction products (lanes with even numbers) and Ni-column purified products (lanes with odd numbers) were separated by SDS-PAGE and stained with CBB. Lane 1 contains a negative control of the cell-free synthesis reaction lacking mRNA.^49)^ Copyright (2002) National Academy of Sciences, U.S.A.

We designed novel enhancer sequences by mimicking the Ω-sequence from tobacco mosaic virus, a 67-base naturally occurring translational enhancer.^46)^ A pool of DNA fragments composed of random sequences (73 nucleotides long) was chemically synthesized in the absence of dGTP in order not to create an undesirable start codon (AUG). These random sequences were inserted behind an SP6 promoter sequence and followed by a luciferase gene as a reporter open reading frame (ORF). The DNA pool was transcribed using SP6 RNA polymerase *in vitro*. The resulting mRNA pool with many different 5′-UTRs was combined with the new wheat germ extract described above, and a ribosome display experiment was performed to select an optimal initiation site for cap-free translation. One of the selected 73-mer sequences showed ∼70% translation initiation activity compared with 5′-capped mRNA, and we named this sequence enhancer-01 of Ehime University (E01).^47)^ We next designed a 3′ UTR element to replace the poly(A) tail, because it was known that the poly(A) tail is dispensable for translation in yeast.^48)^ By varying the 3′-UTR of mRNAs with the E01 5′-enhancer and encoding the luciferase reporter gene, we found that translation does not depend on any specific sequence but solely relates to the length of the 3′-UTR, where about 1,500 nucleotides added downstream of a given gene showed a comparable activity with that of a polyadenylated mRNA^49,50)^ consistent with an earlier report on mRNA stability. Later, we learned that even shorter 3′-UTRs of 150–500 nucleotides possess adequate template activity compared with that of the longer template with the cap structure presented in Fig. 4A. Based on these findings, we constructed a new expression vector, pEU (plasmid of Ehime University, Matsuyama, Japan; Fig. 4B, *left*) to prepare optimal templates for *in vitro* translation. When the DHFR gene cloned into the pEU vector (circular form) was transcribed using SP6 RNA polymerase, three major RNA products were obtained (Fig. 4B, *right*). Assuming that transcription fails at the replication origin (Ori), the lowest band (1.5 kb, about halfway around the pEU vector) corresponded to the expected size from the E01 enhancer, including the DHFR mRNA with the later nucleotide sequence of the plasmid up to the Ori. The middle size band (3 kb) and the upper band transcripts (5.5 kb) were considered to be products of transcription reactions that went around the circular plasmid one and two times, respectively. Of note, subsequent translation assays showed that these mRNAs had practically the same template activity as the one transcribed from a linear pEU vector yielding one RNA product of defined length. We recognized this result as a significant advantage because this may allow skipping of the linearization step using a restriction enzyme, which might recognize additional cleavage sites within the ORF. As shown in Fig. 5A, when a GFP mRNA transcribed from a circular pEU vector was translated in a dialysis bag with our new wheat germ extract, a large amount of protein (9.7 mg of GFP in 1 ml) was produced.^49)^ The translation reaction continued to produce protein for a surprisingly long period, up to 14 days in this experiment. Although supplementation of mRNA was required every second day, the amount of protein produced was higher than that of proteins prepared using endogenous protein synthesis in cellular systems. This result supported our notion that the translation machinery itself is inherently robust and stable. Thus, the pEU vector-based cell-free system is able to produce a large amount of protein products; however, in this form the platform is not yet suitable for high-throughput approaches in the post-genome era.

Meanwhile, we had sought to develop a method to prepare mRNAs in a high-throughput manner for a genome-wide *in vitro* protein synthesis system. A PCR-based method was thought to be less laborious and more cost-effective. In the first attempt, a set of four different primers was used to obtain DNA templates from a cDNA library. The resulting templates consisted of a 5′-SP6 promoter sequence, an E01 element, followed by a given ORF. However, this primer set produced a large amount of short DNA fragments due to primer-dimers, and the PCR products yielded only multiple small RNA fragments in SP6 polymerase driven transcription experiments. Therefore, we designed a set of primers, in which the complete promoter sequence was not contained in any single primer but was only generated when the sequences of two primers were joined correctly (Fig. 4C, *left*), leading to the production of only complete mRNAs (Fig. 4C, *right*).^49)^ We confirmed the mRNA transcribed from these PCR-generated DNA fragments, which showed a similar translation kinetic as shown in Fig. 3C for working with an expression vector. Thus, the split-primer PCR method enables genome-wide construction of transcription templates.^49,50)^

### (2) Development of a bilayer reaction mode for cell-free translation reactions.

Although the CECF method was quite useful for producing a large amount of protein as described above, it was not convenient for genome-wide parallel protein production and subsequent characterization, where a rather small quantity of each protein is preferable in a high-throughput system. To this end, we developed a simple bilayer-based continuous translation reaction mode, which satisfied the principle of CECF reactions, with continuous supply of the substrates and dilution of the byproducts, which may potentially inhibit translation (Fig. 5B, *left*). A similar translation mixture as used for the batch-mode and the CECF reactions was placed at the bottom of the tube, and the substrate solution containing all the necessary ingredients was then loaded as an upper layer with the translation mixture. During incubation, diffusion of the two layers takes place, which follows the principle of a continuous reaction.^51)^ As shown in Fig. 5B (*right*), fluorescence of synthesized GFP was already observed after 5 min, and the reaction continued for 15 hours through slow mixing of the two layers throughout the incubation. The yield of GFP from this experiment was estimated to be around 1 mg/ml of the bottom layer after 15 hours. In Fig. 5C, parallel production and purification of synthesized proteins in the bilayer system is shown. Five mRNAs were transcribed in parallel from PCR-generated DNA templates, in which a sequence encoding the histidine-tag was added at the 3′-end of the ORF. Analysis of each sample by SDS-PAGE clearly showed discrete Coomassie brilliant blue (CBB)-stained bands of the expected molecular weights among endogenous proteins from the wheat germ extract. Purified products obtained from a simple affinity tag purification step are shown (Fig. 5C, lanes 3, 5, 7, 9, and 11). Based on this bilayer mode reaction using a 96-well plate, dozens of proteins can be easily prepared at once. Moreover, co-expression of multiple mRNA species was accomplished,^52,53)^ and several membrane protein complexes were prepared in an active form *in vitro*.^54,55)^ Very recently, a simple protocol to reconstitute chromatin using this bilayer wheat system was reported, in which the mRNAs encoding the four core histones and chromatin assembly factors were co-expressed in the presence of a closed circular DNA.^56)^

**Figure 6.**
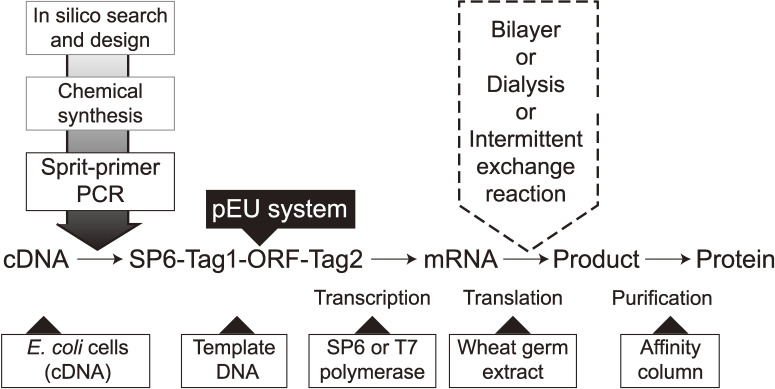
A flow diagram of protein production using the wheat cell-free system. Genes of interest are selected from DNA databases in-silico, and transcription templates can be constructed using cDNAs or chemically synthesized ORFs using the split-PCR method. A typical DNA construct is comprised of an SP6 promoter, E01 enhancer, ORF, reporter, and/or a purification tag (SP6-Tag1-ORF-Tag2). After transcription, the solution is directly used as an input mRNA in the bilayer translation system using multi titer wells. This parallel production system not only allows higher productivity but can also be used in the optimization of the translation conditions (*e.g.*, fine-tuning of ion concentrations, supplementation of a prosthetic group, co-translation with a partner protein(s), selection of detergents for membrane proteins, and incubation temperature) for production of high quality proteins. When one can find a proper protein, move on to the next massive production protocol, from cloning of the construct of interest into pEU and amplifying it to produce a sufficient amount of mRNA, and the translation can be run in CECF mode under a dialysis membrane or intermittent-exchange reaction.

A flow diagram of our cell-free system, combining the elementary technologies described above, is shown in Fig. 6. A comparison of the performance of our system and other conventional cell-free methods is listed in Table 1. It is worth mentioning here that, because the dormant embryos contain few amino-acid-metabolizing enzymes except for two transaminases,^59,60)^ no scrambling of labeled atoms among amino acids takes place during the translation reaction. This made it easy to prepare a stable isotope-labeled protein,^49,61,62)^ and this method has been applied in the recent advent of cellular quantitative proteomic studies.^63–66)^ These methods may provide a procedure for the systematic study of protein structure–function relationships and a series of applications delineating the scope of modern proteomics, including high-throughput enzymatic testing of a large number of genomic expression products, high-throughput crystallization of proteins, and identification of their three-dimensional structure through NMR, cryo-electron microscopy, or X-ray diffraction, rapid evolutionary design of proteins, and construction of technologies for studying protein–protein interactions.

### (3) Automated cell-free protein synthesizers.

**Figure 7.**
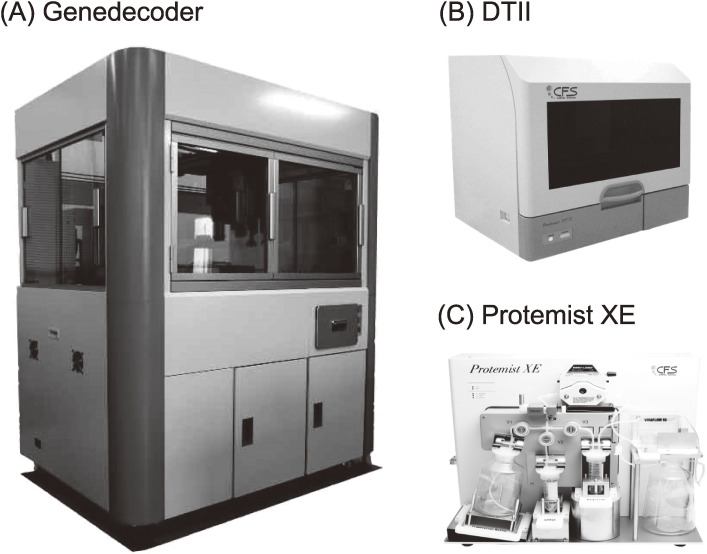
Automated protein synthesizers. (A) Gendecorder. (B) Protemist DTII. (C) Protemist XE.

Combining all elementary technologies described so far, we have developed three types of fully and semi-automated systems (Fig. 7). The Gendecorder has a capacity to synthesize 384 proteins overnight with a small translation reaction size of 150 µl by feeding the template DNAs for the transcription and translation reactions (Fig. 7A). With this technology, a human protein library has been constructed.^67,68)^ The Protemist DTII is equipped with functions for transcription, translation, and affinity purification of up to 6 samples in a medium reaction size of 6 ml (Fig. 7B). Finally, the Protemist XE is for a large-scale protein production (Fig. 7C), equipped with two functions, a repeated substrate buffer exchange (intermittent exchange batch-mode reaction based on the same principle as the CECF) and a continuous supply of mRNA during the translation reaction. Productivity may reach 1 g per 100 ml reaction, and thus is especially useful for downstream applications that require high quality and quantity protein samples, for example for NMR spectroscopy, X-ray crystallography, animal studies, and preparation of antigens.

## 3 From basic research to educational applications

Since first published in 2002, our wheat germ cell-free technologies have been used in various up-to-date research applications such as protein structure and folding, including rather challenging membrane transporters, protein MS, protein–protein interactions, and others, as listed on the website of CellFree Sciences Co., Ltd. (https://www.cfsciences.com/eg/resources/reference-list). CellFree Sciences was established as a venture company of Ehime University to make the wheat germ systems accessible to the research community and to support applied sciences in industry.

Out of the many different applications of the system, our present research initiatives to develop a malaria vaccine are introduced below as an example. Additionally, ongoing outreach to the community based on our wheat cell-free technologies is described.

### (1) Development of a malaria vaccine.

Malaria is a life-threatening infectious disease with a very long history of human infection. Following the emergence of anti-malaria drug-resistant parasites, anti-insecticide-resistant mosquitoes, and the recently emerged multidrug-resistance, malaria vaccine development is a high priority for supporting people in areas where malaria is endemic, and a vaccine may also be of great benefit for travelers from malaria-free countries. Modern malaria vaccine development began with seminal studies in mice using irradiated parasites.^69)^ Decades of endeavor have taught us that achieving this goal will be challenging. One of the problems of malaria vaccine development lies in the preparation of quality proteins from malaria species infecting humans. It is not realistic to prepare malaria proteins from cultured parasites because their hosts are only mosquitos and humans. The first whole genome sequence of *Plasmodium falciparum* malaria was released in 2002, and attempts were made with the use of a cDNA library to produce active proteins using recombinant DNA technology. However, recombinant DNA technology was not successful for template generation, because 1) the codon usage in the genome of the parasite is unique, hence it is essential to adjust codons to match those of host cells such as *E. coli* cells prior to production, and 2) most of the protein products were recovered only in insoluble inclusion bodies. In contrast, the wheat cell-free system was expected to suit the high-throughput preparation of malaria proteins while maintaining high quality. Results from preliminary experiments exceeded expectations, including codon optimization being unnecessary for most of the malaria genes, and the proteins were obtained mostly in a soluble form.^70)^ Currently, a set of malaria proteins has been prepared using the Protemist DTII automated system for the discovery of vaccine candidates with regard to the three life cycle stages, prevention of infection (merozoites in the liver), prevention development of disease (trophozoite in erythrocytes), and prevention of transmission (gametocytes in mosquitos). Very recently, marker proteins for the detection of infection with *Plasmodium vivax* malaria were reported that will hopefully allow the detection of patients with dormant parasites in the liver,^71)^ which until now has been challenging. Furthermore, several vaccine candidates to prevent disease development were discovered.^72)^ A transmission-blocking vaccine is targeting the sexual stage of the parasite in mosquitos and thus blocking the life cycle and preventing the spread of malaria through the community. Tsuboi and colleagues have been working on this dream medicine, and the project is at present at the preclinical stage and soon to be continued with the first in human phase 1 clinical trial.

### (2) Development of teaching materials for local schools.

Besides various contributions to life sciences, I have thought about how to educate students and the general audience on the question of “What is life?”. The basic idea, developed on the basis of what I have learned through my career, is shown below.

“Where Do We Come From? What Are We? Where Are We Going?” These questions asked by Paul Gauguin in the title of his masterpiece have troubled philosophers for thousands of years, and as long as we live, we struggle with them. To the fundamental questions about our existence, René Descartes, who is commonly regarded as the father of modern philosophy, ascertained that “I think, therefore I am”. This principle, which is believed to have formed the foundation of modern science started in the 17th century and appears simple enough to require no further explanation. Nevertheless, I do not think it has provided us with the ultimate answers we have been looking for. Neither am I convinced that philosophical reasoning would eventually lead to an answer to this most difficult, most intimate, yet most remote questions that challenge human beings. I therefore propose that we incorporate life science in the foundations of education in Japan and let young people search for answers to these questions through life science approaches. Living things are all born from parent(s), inheriting their DNA, which encodes genetic information to synthesize proteins. We human beings live and conduct social activities thanks to a vast number of protein molecules – as many as a million different kinds. Life is dictated by the central dogma (general principle) of molecular biology regarding the replication of genetic information and gene expression, which occur through three reactions in the cell, (1) DNA synthesis, (2) RNA synthesis, and (3) protein synthesis. While reproduction of reactions (1) and (2) became possible in a test tube several decades ago, the third reaction (3), namely, making protein synthesis in a test tube, was not possible for a long time. During that time, living cells were the only means for preparing protein samples to study the mechanisms of life carried out by proteins. Then, in the year 2000 at Ehime University, a system of protein synthesis extracted from wheat embryos was successfully adapted to create a practical protein synthesis method. With the advent of this method, protein synthesis in a test tube at will became a reality for the first time. This new technology has been applied for life science research, drug discovery, and medical technology development and has also been used as an R & D tool in other new biotechnology industries. My present proposal is to expand its application to education. The central dogma proposed by Crick about 60 years ago has taught us that life’s activities are carried out through chemical reactions. Although this is widely known and accepted in life sciences, there has been no educational tool available to teach it to laypeople. Such tools can be readily provided by the new wheat cell-free technology. The new technology permits us to demonstrate the life processes described by the central dogma as they are reproduced in a test tube. These educational tools will then help young people to understand logically that all our activities including lofty mental functions are conducted through chemical reactions. Our brain functions do not seem to have evolved to find answers to it through mere thinking; such a capability does not seem to have proved a favorable trait through evolution. Humans need to try things to learn from experience. If so, why should we not enrich education with what life science has accomplished? Visit the website (https://pim-sympo.jp/public_seminar/, in Japanese) for more details.

## Conclusions and perspectives

We have developed a powerful cell-free protein synthesis system by fully using the vital power of wheat embryos. The system has overcome various limitations underling the classical protein production methodologies, in both the cell-free and cell-based approaches. The wheat system converting genetic information into functional proteins in a test tube, solved other serious problems in protein production, namely, biohazardous and ethical issues, because wheat seeds are food products causing no harm to humans. These features have helped the wheat system to make important contributions to drive life science research and technical developments. Since last year, while preparing this review article, we started facing the global COVID-19 pandemic, where once again, our technology showed its value. Our cell-free technology has enabled serological tests for detecting COVID-19 patients.^73–75)^ These examples demonstrated for the first time that our system can be scaled-up to find a suitable antigen and is also applicable to the production of a diagnostic test in an industrial setting.

Although the described cell-free system here can materialize any given mRNA information, the production of active membrane proteins and glycoproteins is sometimes exceptional. As was stated earlier, particular membrane proteins can be prepared in cell-free systems from wheat embryos and *E. coli* cells in the presence of artificial lipid-bilayer.^76)^ However, it is necessary to optimize each protein expression protocol through trial and error, and the success rate is generally low in part due to the highly hydrophobic and amphipathic nature of membrane proteins. Regarding glycoprotein production, N-glycosylation in the form of dolichol phosphate,^76,77)^ our current protocol has limitations due to the lack of appropriate glycosylation apparatus in the system. Our current protocol is able to add the core sugar when the reaction is coupled with a microsome fraction from canine pancreas, although mature sugar chains may not be formed as readily as in other cell-free systems. Recently, Jaroentomeechai and colleagues reported a more advanced methodology using *E. coli* cell-free translation and glycosylation components.^78–80)^ At this moment, we should see how these methods will turn out in the production of glycoproteins. Ideally, efficient systems for the production of these proteins may be realized by adding fractions of endoplasmic reticulum and Golgi apparatus to the cell-free translation system when protocols for the preparation of high quality subcellular components are established.

Important future contributions are envisioned of the wheat system until maybe an entirely different protein synthesis system will replace the ones we know today. Recently, a new concept for protein synthesis using automated flow chemistry has been reported,^81)^ yet it is still challenging to produce folded proteins in a functional form, including membrane proteins and glycosylated proteins, in such a system.

At last, let me draw some perspectives based on our technologies with my personal wishes. Most of the microbes present on the surface of the earth are not cultivable, and it is almost impossible to conduct basic research on such microbes even though they might be highly useful in the biotechnology and industrial sectors. For example, various microorganisms are found as extremophiles in deep earth and deep sea environments, yet most of them are not cultivable in the laboratory. I believe it is possible to materialize genetic information collected by massive meta-genome sequencing datasets from those extreme environments and use protein products made in wheat cell-free systems for studying their biology and biochemistry. This approach may also extend to outer space, where life is currently unknown, but my dreams still reach out to the heavens.

## Figures and Tables

**Table 1.** Comparing the performance of cell-free expression systems^49)^

Materials	*E. coli*	Rabbit reticulocyte	Wheat embryos
Productivity (per ml)	6 mg	µg order	9.7 mg
N-terminal^a^	N-formylmethionine	Mature	Mature
Folding	Post-translation	Co-translation	Co-translation
Quality	Low	High	High
Codon preference	Tight	Tight	Loose
Amino acid-specific isotopic labeling	Difficult	—	Yes
Post-translational modification	No	Yes	Yes
Membrane protein	Yes	Yes	Yes
Disulfide-bond formation	Yes	—	Yes

^a^N-terminal methionine is processed by the endogenous N-terminal methionine peptidase.^57,58)^
